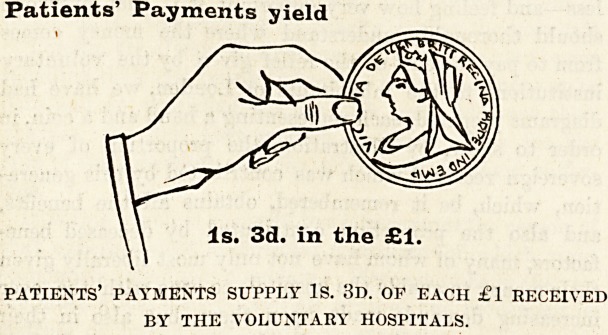# Special Hospital Sunday Supplement

**Published:** 1901-06-15

**Authors:** 


### Special Hospital Sunday Supplement.

### LONDON HOSPITALS OF TO-DAY.

### A VITAL DIFFICULTY.

QUIETLY, silently, unknown to the general public, the managers of several of the older metropolitan hospitals during the last ten years have had to face and overcome a difficulty which at one time threatened the very existence of these institutions. The ordinary owner of a house is enabled by a continuous system of repair, mainly decorative, to maintain it in a habitable and comfortable condition for the indwellers for practically an indefinite period of years, providing the original plan is well thought out and the original work is good and substantially executed. A hospital, however, being a building intended for the treatment of disease, has to be adapted from time to time to the new developments in treatment and to be protected from evils which, previous to Lord Lister's great revolution, made the dangers of hospitalism, that is, the risks incurred by an acute case treated in an old hospital ward, greater even in many cases than the ordinary risks incidental to illness or accident. Matters grew so serious previous to the introduction of the aseptic treatment that in many metropolitan hospitals the mortality after major operations tended to increase until it caused on an average the death of one out of every three people so operated upon. The late Sir James Simpson published statistics many years ago to show that in ordinary buildings operation cases were not liable to this mortality, and that the dangers of hospitalism were proved and incontrovertible. Sir Henry Burdett showed later that in the newer type of hospital buildings like cottage hospitals, the mortality after major operations was relatively unimportant when compared with that which occurred in the great general hospitals. Facts such as those we have been considering induced thoughtful scientists to come to the conclusion that unless a remedy could be found to free surgical cases from the risks of hospitalism, it might be necessary to pull down the whole of the older buildings devoted to hospital purposes, and to reconstruct them on new sites. Then Lord Lister introduced the aseptic treatment, and the results of that treatment are such that the mortality after major operations in our hospitals to-day, is relatively inappreciable. This magnificent result, and the strict enforcement of the doctrine of absolute cleanliness in matters surgical, will cause Lord Lister's name to be remembered for all time as one of the greatest benefactors of the race.

### THE PROBLEM OF THE MANAGERS.

Reverting once again to the difference between a building erected for human habitation and for the treatment of human disease, the latter type of building, if its original parts were constructed, as many of the metropolitan hospitals were, over 150 years ago, was apt to become an aggregate of buildings without any definite plan or any due regard to the purposes to which they were to be put. This result was not unnatural, seeing that, as the population of the district served by a hospital building increased, the hospital authorities had to increase the accommodation for the reception of patients, and so building was added to building, mainly according to the caprice of the particular architect engaged, who was no doubt instructed by the managers in making his plans, to consider how the new buildings could be put up at the smallest cost, and in such juxtaposition to the existing fabric as would render the cost of administration as small as possible. Thus it came to pass that in some of the largest and best hospitals so recently as ten years ago the only communication on the floors above the ground floor, between the wings of which the hospital consisted, was through some of the main wards. In another case we found on inspection that the operation theatre was so situated that in the absence of a lift, patients having to undergo operation were carried through a number of the principal wards on more than one floor to the operation theatre and, after being operated upon, were carried back through the same wards to the bed assigned to the patient. The most thoughtless person will realise the amount of avoidable misery and mental torture which many patients suffered in hospitals of the older type constructed upon the heap of buildings principle from causes such as we have indicated. In the course of years too, whatever may be the original quality of the walls material, or of the substance used for the floors of a hospital, they naturally absorb an amount of deleterious matter and become soft and crumbly in places, so that great risk to the life of the patients may arise from these causes. Thus in due course a time arrived in the history of the older metropolitan hospitals like Guy's, Westminster, St. George's and the London—taking them in the order of the date of their foundation—when the managers who had throughout maintained the external fabric in good condition had to face and to determine the problem presented by the questions of (1) whether the interior could be renewed with efficiency, or (2) whether the whole of the old buildings must be levelled to the ground? Had it not been for Lord Lister and the aseptic system, rebuilding on a new site would have been the only course open to many of the hospitals, and the cost of that alternative would have meant ruin to more than one institution, the very existence of which is of vital importance to the residents in a great modern city. It is not too much to say in this connection that Lord Lister's system has not only saved the hospitals, but has saved the British philanthropic public or the local authorities or the Government—that is to say the ratepayers or the taxpayers as the case may be—a very heavy expenditure indeed for the rebuilding of the older general hospitals all over the country.

### NEW HOSPITALS FOR OLD.

Thanks to the new discoveries the renewal of the interior of the older hospitals, and the addition to them of new sanitary blocks were made possible, and this process of reconstruction has been going on during the last ten years with a zealous regard for the best interests of the suffering inmates, which entitles the managers of the great metropolitan hospitals to the grateful thanks and the appreciative pecuniary support of all classes. Everybody who has a house knows how burdensome the ordinary repairs sometimes prove when they have to be met out of income. It can readily be conceived how great was the responsibility and how real was the anxiety of the responsible hospital managers of the larger London hospitals who had to face an expenditure of hundreds of thousands of pounds in order to modernise the buildings devoted to hospital purposes. It would be wearisome to go much into detail in an article like this, but we believe the preachers on Hospital Sunday at the commencement of the twentieth century can select no more telling ground of appeal to their congregations than to enforce upon the people the knowledge that almost everywhere throughout the metropolis the buildings now devoted to the reception of patients have been so reconstructed and renovated, that quite irrespective of the date of their foundation, the inhabitants of London to-day have the advantage of possessing new and modern hospitals which cannot be surpassed for efficiency and completeness. It speaks volumes for the public spirit of the hospital managers, and entitles them to be regarded as worthy upholders of the fair fame of the metropolis of the Empire, that when deciding to make the great hospitals new and modern they also determined to provide everything in addition which modern science had proved to be essential to the rapid and speedy cure of the patients. Thus we find that there is hardly a hospital in London which has not in hand, if it does not already possess, a block of buildings specially designed and apart from the hospital buildings proper for the adequate accommodation of the nursing staff. As the population of London has grown, and the tendency to crowd more and more human beings into less and less space has increased, the liability to accidents and casualties on the part of young and old, among the poorer classes especially, has grown to an extent the average Londoner has no conception of. To cope with this added volume of pain and suffering due to minor casualties arising mainly from the overcrowded condition of the dwellings and streets in the poorest neighbourhoods of London, the great hospitals have established a new organisation and modern accommodation for the treatment of these cases, which have involved the erection of huge examining rooms and extensive day surgeries with small consulting rooms attached, where this type of patient is promptly attended to by a special staff who are always on duty throughout every period of each twenty-four hours. Who can assess the immensity of the saving in human suffering alone which these expensive and modern improvements have resulted in?

Three-quarters of a Million Well Spent.

Coming now to figures, and taking only the four hospitals mentioned as typical of other London institutions, we find that the expenditure rendered necessary to renew the old buildings and to meet fully the needs of the present day, amounts in the case of Guy's Hospital to £180,000, the Westminster Hospital to £68,000, St. George's Hospital to upwards of £100,000, and the London Hospital to £370,000. It will thus be seen that, without any fuss and with a courage which is worthy of public recognition, within the last twenty years the authorities of these four great and ancient hospitals have entered upon a liability of nearly three-quarters of a million pounds sterling, in order to modernise their hospital buildings and make them models of what such buildings should be, having regard to scientific developments and the paramount importance of diminishing the stay of each patient in the hospital. This surprising fact will have increased emphasis when we add that a sum of upwards of £500,000 sterling represents the actual expenditure upon improvements and renewals which these four hospitals have decided to undertake within the last ten years. Is it to be believed, seeing, as we shall prove in our next article, that the cost of treatment each year necessarily grows, that the inhabitants of London will be content in the first year of the twentieth century to contribute on Hospital Sunday a less sum to the hospitals than £50,000.

### The Gain to the Breadwinner: The Cost to the Hospital.

Two Points of View.

There are two ways of approaching the question of voluntary hospitals on Hospital Sunday. First of all they should be regarded from the point of view of the patient; and secondly, from the point of view of their cost and the value of such expenditure to all classes of people, whether rich or poor. First as to the patients: the huge mass of human suffering alleviated, of disease cured, of preventible miseries lessened, who can estimate? To these considerations must be added the gain by the removal of the breadwinner from surroundings where treatment would be dangerous and must retard recovery, if they did not militate altogether against cure, to one of our great palaces of pain where everything requisite to promote speedy recovery is provided, regardless of cost and from the sole point of view of the best interests of the sufferer. In the previous article we have shown that it has taken hundreds of thousands of pounds to equip and render as nearly perfect as possible for their object the great London hospitals. But with improvements in fabric, in nursing, in fittings and furniture, and in the quality of dressings and drugs there has grown up a steady increase in the expenditure, which mainly arises from a shortening of the stay of each patient in a hospital bed, due to the rapidity with which recoveries are effected under modern hospital treatment.

Sixteen Working Days Gained.

What this additional wage-earning power represents to the individual patient may be shown by quoting a few figures from the London Hospital at Whitechapel. In 1890 the total number of in-patients treated was 9,193; in 1900 the hospital returns show that 12,154 persons were treated as in-patients—that is to say, the increase in the number of in-patients in ten years, though the available beds have been nearly a constant quantity throughout, has increased by 33 per cent. We merely quote this instance because the figures are before us, but it must be understood that similar results have been achieved by the other great hospitals where the management has shown equal intelligence and public spirit. The great and glorious fact which underlies the possibility of treating 33 per cent. more in-patients in a given number of beds than was the case ten years ago, is due to the rapid cures effected by improved methods of treatment and perfect hygienic surroundings, which have resulted in reducing the average number of days during which each in-patient was resident in the hospital from 35 days in 1880 to 23·87 days in 1890 and to less than 20 days' residence in 1900. This reduction in the days resident is due to the better and more favourable circumstances under which patients can now be treated, to the supply of modern appliances for the use of the medical officers, and last, though not least, to better nursing. Here is a text for every preacher, for who can over-estimate the advantage to the working classes, and through them to the whole community, of returning the breadwinner who is struck down by disease, or overtaken by accident, well and sound to his work after a residence in the hospital of 19 days, compared with a stay of 35 days in hospital so recently as 20 years ago. We would go further, for it cannot be doubted that, whereas the improved sanitary conditions and methods of treatment have curtailed the residence of the working men in our hospitals by 16 days in present circumstances, the fact of these extra 16 days of residence under less favourable circumstances of hygiene and treatment must have materially affected the strength and the working powers of every patient who suffered it. The whole community are surely under a deep obligation, and no one with a heart—and it takes a heart to move a body—can deny the obligation, to give to the voluntary hospitals on Hospital Sunday.

### A Magnificent Result.

Let us recall the facts for a moment. Thanks to the great teacher, Lord Lister; to the public spirit of the hospital managers who have made our hospitals what they are to-day; to the devoted zeal and scientific attainments of the medical staff; to the tender care of the nurses, residence in a hospital has become one of the most important factors in the economy of the nation. Every inmate of a hospital under modern conditions, taking the average of all the in-patients treated at the present moment, gains through the development in modern hospital treatment 16 clear working days, and the wages which result therefrom for the benefit of his family, on each occasion when illness or accident may render it necessary for him to seek the aid of the hospital. There were no less than 98,000 in-patients treated in the hospitals and medical charities of London during last year, so that on a moderate computation the solid benefit in pounds sterling resulting from the efficiency of our voluntary hospitals to-day, as compared with 20 years ago, may be realised by remembering that it added over one million working days to the earning powers of the artizans of London alone. We have been emphasising the practical side of this particular point of modern hospital development, and we leave it to the preacher, as a man who has sound knowledge of human suffering and of all the trials which affect humanity, to bring out the humane aspect of this magnificent result secured by our great medical institutions at the commencement of the twentieth century.

### The Trained Nurse.

Take another point, from the patient's aspect—the trained nurse. At Guy's Hospital—and the system was the same at any great hospital 30 years ago—the whole staff of nurses amounted to 72, and that was an increase of nearly 50 per cent. on the number employed some 12 years earlier. At that time, with the exception of an allowance from the hospital of bread and beer, the nurses boarded themselves and provided their own clothing. In most hospitals then the ordinary nursing staff was supplemented by the engagement of night attendants of the scrubber class, who received 10s. a week for acting as so-called night nurses; though in reality, as the writer knows from his personal inspection of the hospitals in those days, they took this money for the privilege of sleeping in the hospital ward instead of sleeping in their own beds at home. To-day the nursing staff of Guy's Hospital numbers 231 persons, exclusive of wardmaids, dormitory maids, and others. All these nurses are boarded entirely at the expense of the hospital, and are provided with uniform and everything essential to promote their health and conduce to the efficiency of their work. It will be noticed that the increase in the numbers of nurses engaged in a hospital nowadays is nearly five times the number employed formerly: it illustrates at a glance the huge development which has taken place in the nursing of the patients and the care of the sick; it shows how much the hospitals appreciate the importance of the closest attention to the minutest instructions of the doctors in the treatment of individual patients; and a word of acknowledgment is surely due to the managers, and to the devoted men and women who constitute the heads of the resident staff of the hospitals, for all who know hospitals well can appreciate the difficulties involved in the proper control and training of such a large staff of nurses, however much may have been done to improve the accommodation by the provision of a nurses' home of the most modern efficiency. It is an axiom those who know most about sickness will uphold, that nurses well cared for mean patients well cared for too. Every legitimate improvement in the condition of the nurses immediately reacts to the benefit of the patient. Hence the very large expenditure which has been entailed by the introduction of modern methods of nursing has had a material bearing upon the cure of each patient, and that bearing will be emphasised in every hospital where an adequate provision is made for the nurse to live a wholesome life mentally and physically, in which connection not the least important feature surely is the provision of a separate bedroom for each member of the staff.

### Modern Developments in Treatment.

Once again, the patients benefit by the new methods of treatment and the increased facilities for the performance of the enormous number of surgical operations which modern science has made available. Take, for example, the Finsen light for the treatment of lupus, one of the most shocking and disfiguring form of disease to which the human race is liable. At the instance of our noble Queen Alexandra, at that time Princess of Wales, and rightly known and beloved as "The Princess of Pity," a Finsen light was established at the London Hospital similar to those which had been tried in Copenhagen with the greatest possible success. In the result a large number of patients whose cases were formerly regarded as incurable have been restored to health and saved from the effects of a most loathsome and shocking disease. The discovery of the X-rays opened a new vista in surgical treatment, and is now everywhere employed, to the enormous advantage of the patients. In a large hospital the X-rays entail the employment of a busy room, with five or more apparatus constantly at work in charge of skilled and trained assistants. Photography now takes an important place in the work of a large hospital, and the photographic department renders yeoman service to the medical staff by recording and illustrating the cases under their charge. The sterilisation of all the instruments, dressings, and ligatures used in the theatres and wards has developed year by year with the progress of knowledge, and with the opening of new and improved operation theatres, with their necessary anæsthetising rooms and instrument-sterilising rooms attached, the management of every great hospital is alone able to meet all the demands of modern surgery so as to secure the utmost benefit to every patient admitted to the institution. We have thought it well to indicate in detail these various modern developments in treatment, because, though medical knowledge increases steadily, its progress is necessarily not definitely marked by lay observers, the majority of whom have probably never heard of the Dowsing bath for the treatment of patients by heat generated by electric light, or of the Tallerman bath for the hot-air treatment of rheumatoid arthritis, and so do not realise how large is the number of cases, not only of in-patients but of out-patients, who secure the advantage of these appliances; nor the great and increasing number who are each year treated at the hospitals by massage to prevent stiffening of the limbs, for which a decade ago little or nothing could be done. It surely requires but a small amount of imagination to picture the immensity of the measure of relief from suffering nowadays afforded in our hospitals to the less fortunate members of our race. Yet the excuse often made by people who in God's providence have hardly known what it is to suffer, though blessed with wealth, when asked to give liberally to a hospital, points to the conclusion that the difficulty of the hospitals, so far as money is concerned, has its chief origin in ignorance of the true meaning of their work and of the splendid way in which they accomplish all that is possible for the good of every class of the community.

### The Cost to the Hostital.

Turning next to the cost of these splendid results, we shall content ourselves with pointing out that all these improvements, all this gain to the Earning power of the working classes, all the lessened suffering, and all the added comfort means increased outlay in money. Thus, considering the position of each individual patient, it will be manifest that a person acutely and continuously ill must require closer attention and special and expensive treatment which, whilst they diminish the number of days of hospital residence, materially increase the cost of each in-patient nowadays as compared with 10 years ago. We have worked out the figures and find that the average cost per in-patient has increased by something like 10 per cent., and that, whereas the average cost per in-patient 10 years ago was something like £5 per patient, it is now something like £5 10s. This fact hits the hospital in two ways. First of all, we find that a fixed number of beds in a hospital 10 years ago enabled 9,193 patients to be treated; and that to-day, under modern conditions, the increased cost of treatment would be upwards of £4,500 per annum. But these improved conditions lessen the days' residence, and so increase the number of in-patients from 9,193 to 12,154, the number which it is now possible to treat in the fixed number of beds. Hence the actual cost to a large hospital is not £4,500, but upwards of £6,000; but the community has had the additional advantage each year of securing the adequate treatment of 3,000 more in-patients in the same number of wards and the same number of beds.

Again, the development in trained nursing has entailed a very large outlay upon buildings of, say, from £15,000 to £20,000 in the case of any of the larger hospitals, with a very material increase in the annual expenditure, due not only to the fact that the numbers of the nurses have increased, as we have already shown, more than threefold, but that there have been other and heavier additions to the expenditure owing to the improved system of nursing entailing the employment of a large number of additional ward and dormitory maids and other members of the staff. It would be wearisome to go minutely into the details, and it must suffice for the present purpose to say that the actual annual increase in the expenditure of a large hospital due to the improved methods of treatment and the new payments which have had to be met, is equal to an increase of about 25 per cent. of the total expenditure. The expenditure of the London hospitals during the year 1900, as enumerated in the tables on pp. 17–20, amounted to £814,284. It will thus be seen that the hospitals are entitled to receive from the public in direct contributions each year not less than £200,000 to reimburse the necessary outlay entailed by the improved methods of treatment which, in the case of every workman in-patient treated each year, adds 16 working days to his earning capacity, and so brings increased comfort and happiness to his wife, his children, and himself.

### An Ideal Realised.

It has always been the aim of the most experienced and ablest hospital administrators to make their hospital cure the greatest number of patients in the fewest number of beds in the shortest possible time. We have shown in the course of this article that that aim, so great is the development in the unit of hospital efficiency everywhere in the present day, is being successfully fulfilled in every one of the great metropolitan hospitals. Shall it be said that the citizens of the metropolis of the Empire at the commencement of the twentieth century were so blind and indifferent to the splendid work of the hospitals, and to the claim which they have upon rich and poor alike, that they were content to contribute less than £50,000 on Hospital Sunday, 1901? May we not rather hope in these days of education that the average of intelligence is so high in this country—and especially in London, the heart of the Empire—that through the influence of the preachers on Hospital Sunday there will this year be an awakening and quickening of the sense of responsibility of each individual citizen, so that the sum raised directly for the hospitals on Hospital Sunday, the 16th instant, will at least amount to £100,000, whilst the indirect gain to these noble institutions will, within a reasonable period, be adequate to repay to the hospital exchequers the whole of the hundreds of thousands of pounds which have been so wisely expended in modernising our Metropolitan hospitals. It is, however, the first object of the Hospital Sunday Fund to raise the money which has been expended upon improved methods of treatment resulting in the immense gain to the working population, and indeed to all classes of the population which we have proved to have resulted from this wise outlay of charitable funds.

### Preachers and Hospital Sunday.

From fulness of knowledge and a belief in the vitalising influence of a preacher who speaks from personal observation, we would venture to urge every clergyman and minister of religion who will occupy a pulpit on Hospital Sunday, 1901, to make a point of visiting either the London Hospital, Whitechapel, or Guy's Hospital, which is close to London Bridge Station, before Hospital Sunday the 16th instant. Dr. Perry, of Guy's Hospital, and Mr. G. Q. Roberts, of the London Hospital, would extend a hearty welcome to every such visitor, and we would suggest that each preacher should see for himself the casualty department, so that he may understand the work done there, the photographing and Roentgen rays rooms, the Finsen light room, and that a careful inspection should also be made of one or two of the renovated wards. If circumstances should prevent a visit to either of the two hospitals mentioned, then let the preacher or pressman call at the hospital which is nearest to him in his own district and ask the authorities there to show him such portions of the institution as will give him an insight into its actual work and progress. We urge the preachers to put themselves into the sermons they will preach on Hospital Sunday, 1901, for then the hospitals will undoubtedly reap a golden harvest worthy of the first Hospital Sunday of the Twentieth Century.

### The Ever-changing Art of Medicine.

It is the lack of finality in medical science which is to a large extent responsible for the absence of all finality in hospital construction and for much of the expense of hospital treatment. If we could be content that hospitals should retire from their present position in the van of progress certain small economies might probably be effected. But then we might as well have a row of workhouse infirmaries which we already have as by law established. The real excuse for the existence of our great voluntary hospitals is that they give something that the legal hospitals of the country, the workhouse infirmaries, cannot give, namely, the services of the very cream of the medical profession and treatment which is at the very growing point of medical progress.

We have spoken of medical science as being the spur in response to which so much expenditure is undertaken by the hospitals for the benefit of their patients, but in truth the term medical science is rather a misnomer. If medicine were a science such as physics or astronomy, founded upon well-known laws, fixed and immutable, all these changes would not be. But medicine is not a science in that sense of the term. It is founded upon science; at every new development it harks back to science for confirmation or refutation; and the discoveries of investigators in every field of science are constantly being turned over by physicians in search of something which may perchance prove useful to their patients. But in its daily application to the cure of disease and to the alleviation of suffering, medicine is essentially an art, a constantly progressive art, and one which, like other arts, admits of no finality, and always struggles forwards and to higher planes. Hence all the troubles, hence these constant changes in hospitals, hence all this expense, and as a set-off hence all these cures in regard to which each succeeding decade says, Who would ever have believed ten years ago that such things could be?

Think of the discoveries which have been made in medicine, and even more in surgery, within the memory of men still living. It is not yet sixty years since the discovery of anæsthesia. Up to that time an operation, a thing which is thought of so lightly nowadays, was a thing of horror unutterable; an ordeal only to be undergone by the patient when at the last extremity; a thing so revolting to all concerned that it was only undertaken by the surgeon under the strongest sense of duty. The introduction of anæsthetics changed all this—it absolutely removed all the old terror—and surgery at once entered on a new phase. Perhaps we who have never seen operations done, as in old times, on the sentient, shrieking, struggling patient, whose whole frame quivered at every movement of the knife, do not appreciate to the full the blessings of anæsthetics in regard to the saving of pain; but it is certain, on the other hand, that the men of those days did not foresee or appreciate what a blessing anæsthesia would be in extending the field of surgery. All kinds of diseases are now cured by the knife, simply, painlessly, and completely, in regard to which the very thought of such interference would never have occurred, either to surgery or to patient, so great was the reluctance to endure or even to suggest the torture of operation. The older surgeons measured the blessings of anæsthesia by the relief it gave to pains which otherwise would be unavoidable. But the blessings of the anæsthetics which are administered scores of times every day in our great hospitals are really to be measured by the fact that they have rendered it possible to bring surgery to the aid of a host of conditions which otherwise would have been allowed to drag out their weary days uncured. And this is how anæsthetics have reacted on the hospitals: they have doubled their work—nay, they have increased it tenfold. What to the individual, and indeed to the public, has appealed merely as a means of easing and preventing pain has to the hospitals meant increased work, new operating theatres, a larger staff, a greater number of dressers; operations going on morning, noon, and night; expense, expense, expense, if the hospitals are to do their duty and to give to each patient coming to their doors the best they can.

Just the same thing has occurred in regard to antiseptics. Of the life-saving efficacy of Lister's great discovery no one could doubt. Hospital managers in the early days grumbled at the expense of the treatment, but the lives were saved, and that was a great thing. As was the case with anæsthetics, the benefit of the new discovery was at first measured by its life-saving effect upon the then existing number of operations. In a very few years, however, it was seen that the effects of antiseptic methods were to be measured not by what they did for the operations which were then being done, but by the extent to which they rendered possible other operations, the very nature of which had never even been dreamed of. Again, it happened that the very invention which was showing itself to be of such incalculable benefit to humanity was well nigh the financial ruin of some of the hospitals. From the early days the dressings were ruinously expensive, compared with those previously in vogue, and as the real foundation of the system came to be better understood, and as it became evident that the fight against the germs of disease must not be confined to the seat of operation, but must be extended to the doctor, to the nurse, to the bed, to the ward, the operating room, and the whole hospital, so did the expense grow greater still. Although the dressings now used are as simple as may be, the total expense of conducting a surgical hospital has become even greater than ever. In return we have all the triumphs of modern surgery of the benefits of which the hospital patients have always been the first to partake. But the very success of modern surgery which has so popularised it has at the same time thrown an enormous strain upon the financial resources of our hospitals. It has perhaps saved them from being pulled down, but it has vastly increased the expense of carrying them on.

In the medical art something new is constantly arising. One year we have X-rays, another hot-air baths, another serum treatments of varying efficiencies, and so on. Not always an advance along the true line—like a line of skirmishers sometimes pushing forwards, sometimes rushing to a point of vantage on one side or the other, sometimes even for a time beaten back, but still on the whole advancing—so does the medical art go on, and so must the hospitals also if they are at all times to do the best for their patients. It is useless then to look for finality, or to expect, in business phrase, that the capital account shall be closed. New circumstances are constantly arising and new discoveries being made, and the hospitals have to do the best they can to give the sick poor the benefits they offer. Hence much expense and many calls on the charity of the public.

No more striking example of what is constantly happening in hospital management could be given than the consequences of the introduction of Finsen's "light treatment" at the London Hospital. The London Hospital is an institution which for the past 160 years has been of incalculable service to the denizens of the eastern portion of the metropolis. By great good fortune or by happy foresight its founders so placed it as to make it possible to extend its buildings from time to time as need arose, and with the growth of London time after time has this need arisen. No institution in the metropolis has striven more usefully or with greater energy than the London Hospital to meet the wants of the teeming population of workers by which it has been surrounded on every side. Five or six times within the last half-century has the hospital been enlarged, and even now it is still being enlarged. As the land which is available has become more and more covered, it seemed that perhaps at last the finishing touch would be given, and that this great institution would soon be "rounded off" as a complete establishment for the cure of disease. Vain expectation. The light treatment came along. It was impossible for the managers to refuse to the poor at their gates the benefit of a treatment which was known to be of such marvellous efficiency. Queen Alexandra, then the Princess of Wales, interested as she was in things from Denmark, and interested as she always has been in all that tends to lighten the lot of the sick poor, saw this treatment at work in Copenhagen, and presented the London Hospital with a set of the apparatus required for its installation, and the "treatment" proved a marvellous success. It has been a great thing that the poor of the East End of London should thus have access to so potent a cure for so frightful a disease as lupus, and they have shown that they have appreciated it. But there has been such a "run" on the light treatment that it has been quite impossible to meet all the demands, or to give it to all the applicants. What then about finality? and what about rounding off the London Hospital as a complete and completed institution? New lamps for the treatment have had to be added, more rooms have had to be devoted to the work, doctors and nurses have had to be detailed, and, in fact, an expenditure of about £400 a year per lamp has had to be undertaken in order that the managers of the London Hospital may fulfil their trust and may provide for the poor the best possible treatment for their diseases. And this is but an example of what is happening all around. Clearly, if the hospitals are to do what the public demands that they shall do, the public must also do its duty and must put its hand deep into its pocket to pay the bill, for all these special forms of treatment are desperately costly.

### The Cost of the Hospitals.

Hospital Sunday is once again close upon us, when the Churches will endeavour to concentrate the thoughts of Londoners upon the beneficent work which is being done by the hospitals, and to urge upon them, in the name not merely of charity, but also of humanity and of good will towards their fellows, to give freely towards the support of institutions which do so much for the relief of the suffering which exists on every side.

In putting forth this appeal to the liberality of our fellow Londoners we cannot but think that at a time when progress is so much the watchword as it is at present, when London is waking up in response to ideas which have long been active forces in other cities, and when everyone feels the tingle of that ambition to be in the forefront which is at the root of most great deeds, it is well that we should point out that one of the great claims of the hospitals of London upon our support, and indeed one of the very reasons why they have so often to come hat in hand begging for charity, is that the hospitals of London are always progressive, and that they are always spending their last penny in keeping their appliances up to date and in providing for the sick poor, who are their charges, the best that modern medicine offers for the cure of their diseases and the alleviation of their sufferings.

It is this which is no doubt to a large extent the reason of that expensiveness of hospital treatment of which rigid economists sometimes complain. If we are to be in the van we must always move, and all change and movement in matters of bricks and mortar is expensive. Those who have watched most carefully the gradual evolution of ideas in regard to hospital construction are the most ready to maintain that although now and again false moves may have been made, and although results have not been quite equal to anticipations, everywhere the motive has been the same, and the reason for the somewhat lavish expenditure which has taken place from time to time in hospital construction has been the desire to give to the sick poor the full advantage of the most advanced medical science of the day.

Time was when hospitals were refuges for the destitute rather than of cure for the sick. In days when the poor had no rights, when man lived solely on the fruits of his own labour, and when so soon as sickness laid him low he had to depend for very life on the fickle offerings of casual charity, then a *hospice*, a mere refuge where he could lay his head, was a charity indeed. Nothing enables one to realise more vividly the terrible condition of the sick poor in days gone by than the descriptions given of the accommodation then provided by the hospitals—hospitals in which treatment was out of the question, hospitals whose offer of a roof, a crust, and a third or fourth share of a bed was sufficient to draw crowds of applicants.

It is curious that from almost the beginning war has been the great incentive to hospital reform. The great wars that marked the turn of the century a hundred years ago greatly altered the uses to which the Continental hospitals were put. Instead of being what we should call workhouses they became crammed with sick and wounded soldiers, and it quickly became apparent how unfitted they were in many cases for their new *métier*. They became pest-houses from the development of special, so-called, "hospital diseases," diseases due to the aggregation of masses of sick under the conditions which then prevailed. It quickly became evident that if the sick were to be safely brought together in considerable numbers the buildings must be specially arranged, and thus hospital construction became to a certain extent a special branch of architectural work—one, however, which at first was but ill understood, and the mistakes in which have had to be heavily paid for over and over again.

Again the Crimean War, with all its horrors, at least acted as a great hospital reformer, and under its influence hospitals of a better type covered the land, again representing the position of the knowledge of the day. But knowledge declines to stand still. With new knowledge new types of hospitals came into vogue, and hospital managers in their desire to do the best for the sick under their care had to make alterations and to spend money in so doing. This seems inevitable; finality is unattainable. The teaching of the great wars, in the beginning of the century, in the Crimea, in 1866 and in 1870, was all to the effect that not many sick people should be under one roof. There was some mysterious and baneful influence about the aggregation of invalids which was spoken of as "hospitalism," and opinion drifted all in favour of putting up numerous separate pavilions instead of massive buildings for hospital purposes. There are doubtless hundreds of hospitals, which have cost hundreds of thousands of pounds, built under the influence of what was then the best knowledge of the day. And very good hospitals they are. But since the days of the Franco-Prussian war antiseptic surgery has dominated the position, and new developments in hospital construction have made themselves felt, and still more are threatened, which may make the hospital of the future a very different affair from that of to-day. There is no finality. The model modern hospital becomes representative of what is past almost before its paint is dry, and further expenditure becomes inevitable.

Why not let well alone? say some. But right as that may be in regard to some things, it cannot be done in regard to hospitals. In hospital affairs the difference between "well" and "very well" means lives lost or saved; an improvement of so many per cent. in the mortality of a hospital means that so many per cent. of its patients are now living who in a less well managed hospital would be dead; and if we place ourselves in the position of hospital managers with the responsibility for all these lives placed in their hands, we cannot wonder that hospital treatment sometimes appears expensive, or that those responsible for hospital expenditure should feel that in asking for monetary help from a generous public economy is not everything, and that their best excuse and their most effectual plea when making their yearly request for more and still more money is that more and still more lives are being saved by the hospitals being always kept well in the van of progress.

### A Word to Living Londoners.

### Ways and Means.

When giving these statistics for 1898 in our Special Hospital Sunday Supplement last year we said that, although the figures from which to form a complete analysis for 1899 were not available, it was apparent that there was a falling off in the amount received from charitable or voluntary contributions towards the care of the sick in the metropolis. A comparison of the figures given below with those published last year will prove how correct our forecast was. It will be seen that, including St. Bartholomew's Hospital, the living—namely, the present inhabitants of London—gave 8s. 7d. in the pound of the total income received by the London hospitals in 1899, which added to the 1s. 3d. received from patients' payments, only brings the contributions of the living up to 9s. 10d. in the pound, as compared with 10s. 7d. in 1898. A moment's reflection must convince us that this is altogether an inadequate proportion for the living to give. It shows that our great London hospitals are not supported either by the patients who are so ready to avail themselves of the benefits to be obtained from them, or even by those who subscribe to them, but are in reality supported up to over one half of their total receipts by the charity of those who have gone before. It is certainly not a state of affairs for the present generation to be proud of, and should make us realise that it is our bounden duty, individually, to do all in our power on behalf of the medical charities of London, and not only to increase our own contributions to the Hospital Sunday Fund, but urge others to do the same.

### The Income Available for the Work Done.

In the year 1899, exclusive of the fever patients treated at the hospitals of the Metropolitan Asylums Board, hospital treatment was provided for patients numbering about one million and three-quarters, and the total income which the London voluntary hospitals and dispensaries received during the year 1899 for this purpose was £1,097,776, which was derived from the following sources:—

| | |
|---|---|
| Charitable or voluntary contributions ... ... ... | £472,590, or 43%. |
| Income from invested property | 274,552, or 25%. |
| Legacies ... ... ... | 280,913, or 26%. |
| Patients' payments ... ... | 69,721, or 6%. |

So far as the above figures refer to St. Bartholomew's Hospital, they have been confined to that portion of the revenue which was applicable to hospital purposes.

### How the Money is Provided.

We are quite aware that to many people a mere sequence of figures and percentages is troublesome and often unconvincing—indeed, may to some be even meaningless—and feeling how very important it is that everyone should thoroughly understand where the money comes from to pay the cost of the relief given by the voluntary institutions to the inhabitants of London, we have had diagrams prepared, each representing a hand and a coin, in order to show, by illustration, the proportion of every sovereign received which was contributed by this generation, which, be it remembered, obtains all the benefits, and also the proportion contributed by deceased benefactors, many of whom have not only most liberally given their money to enable the hospitals to cope with the ever increasing demands made upon them, but also in their lifetime took an active part in the management of these institutions. With a view to clearness and ready comprehension the diagrams have been drawn to scale to represent the proportion of every sovereign given in 1899 by (*a*) the dead, (*b*) the living, and (*c*) the patients themselves. *The black hand* and the coin held by it represent the contributions from those now dead; *the white hand* represents the charitable contributions of the living, *i.e.*,

**The Dead Hand gave**

**10s. 2d. in the £1.**

THE DEAD HAND GIVES 10S. 2D. OUT OF EVERY £1 RECEIVED BY THE HOSPITALS.

ourselves, the living Londoners; and the smallest coin indicates the amount received from patients' payments.

Of every sovereign received 10s. 2d., or over *one-half, is*

**The Living gave**

**8s. 7d. in the £1.**

THE LIVING, I.E., THE PRESENT INHABITANTS, GIVE 8S. 7D OF EACH £1 RECEIVED BY THE HOSPITALS.

*derived from legacies and the interest upon gifts* from deceased benefactors, which have been invested in approved securities; 8s. 7d. out of every sovereign has been *given*

**Patients' Payments yield**

**1s. 3d. in the £1.**

PATIENTS' PAYMENTS SUPPLY 1S. 3D. OF EACH £1 RECEIVED BY THE VOLUNTARY HOSPITALS.

*in charity* by the present inhabitants of London—that is *the generation for whose benefit the money is being spent:* and 1s. 3d. of every sovereign received has been contributed by *those who have been actually under treatment in the hospitals.* It will be well for us to consider these facts and study these figures carefully for a moment. The first point to claim our attention is that a very considerable portion of the 10s. 2d. is derived from legacies received during 1899, and it must be remembered that this source of income is a fluctuating and unreliable one. It happens that during the last two or three years the amount received from this source has increased each year, but we have only to go back to 1896 to find that the income received from legacies suddenly fell about £40,000 below the amount received in 1895, and the same thing may happen again at any time, bringing us face to face with a deficiency which, unless the contributions of the living are steadily increased each year to avoid such a contingency, would seriously cripple the work of the hospitals. We have no right to trust to the dead hand, which may fail us not only for one year but for several years in succession. It is our duty, it is only common honesty, to be up and doing ourselves.

THE MEANING OF THE DIAGRAMS.

The foregoing figures deserve the careful attention of all classes in London. They show that although the people in London make use of the voluntary hospitals in greater numbers than the population of any other city in the United Kingdom, they are satisfied with paying under half their total cost, and are content to trust to the dead hand to supply the deficiency. In the provincial cities of importance the greatest pride is taken in the hospitals, and all classes combine to provide the necessary funds. Surely it is not too much to ask that we should emulate their good example.

The ever-growing demand upon the resources of the hospitals and the rapid extension of the population of London are facts which speak to us with no uncertain voice. Those who take any personal interest in hospitals, or study the reports of their work, know how many of them have beds, sometimes whole wards, unoccupied for want of funds. Were it not for the contributions of the dead hand many would have to be closed altogether. Year by year the number of the sick poor to be cared for must of necessity increase, and unless a sustained effort is made to meet the demands we Londoners, proud citizens of the richest and most powerful city in the world, will have to smart under the reproach of being so careless, so selfish, as to grudge giving adequate help to our suffering and less prosperous brethren. We repeat that the effort must be an individual one: we must not leave it to the rich man, nor even to our next-door neighbour who happens to be a little better off than we are. To plead inability is, in nine cases out of ten, mere dishonesty. We can all do something; we can all give our mite; and since we have a sure knowledge that it is more blessed to give than to receive, he will never be the poorer who gives willingly and unstintingly towards the care and comfort of the sick, the suffering, and the dying.

This, the first year of a new century, is surely a time for making good resolutions. We who are in health, in prosperity of a greater or less degree, have our responsibilities which we may not neglect. The care of the sick is not a matter we can relegate to others, it is our affair—rightly considered, our privilege. Let us, then, not only as a duty, but as a thanksgiving for the blessings which we enjoy, make up our minds to give freely on Hospital Sunday, so that there may be no check in the great work which our hospitals are doing.

### Metropolitan Hospital Sunday Fund, 1901.

### A YEAR'S WORK IN THE HOSPITALS AND MEDICAL CHARITIES OF LONDON.

### NEWINGTON AND SOUTH DISTRICT.

Comprising Battersea, Wandsworth, Tooting, Balham, Streatham, Brixton, Lambeth, Newington, Southwark, Bermondsey, Camberwell, Greenwich, Deptford, Lewisham, Blackheath, Woolwich, &c.

| No. of Beds. | No. of Beds Daily Occupied. | HOSPITALS. | In-patients. | Out-patients. | Total Expenditure. | Income. Charitable. | Income. Proprietary. | Income. Patients' Payments. | Total Income. | Legacies not included in previous column. |
|---|---|---|---|---|---|---|---|---|---|---|
| | | | | | £ | £ | £ | £ | £ | £ |
| 650 | 464 | Guy's ... ... ... ... ... | 6,887 | 104,683 | 95,404 | 13,804 | 30,962 | 4,035 | 48,801 | 13,087 |
| 11 | 6 | Phillips' Memorial Homœopathic | 93 | 2,436 | 711 | 368 | 152 | 175 | 695 | 45 |
| 25 | 23 | Miller ... ... ... ... ... | 309 | 16,532 | 3,756 | 2,655 | 371 | ... | 3,026 | 1,250 |
| 269 | 226 | Seamen's ... ... ... ... | 2,400 | 23,192 | 19,270 | 8,001 | 3,761 | 660 | 12,422 | 6,172 |
| 569 | 419 | St. Thomas's ... ... ... | 5,821 | 62,361 | 68,383 | 6,255 | 49,644 | 293 | 56,192 | 2,904 |
| 66 | 59 | Evelina, for Children ... ... | 1,060 | 7,025 | 7,514 | 3,185 | 3,446 | 106 | 6,737 | 20,732 |
| 57 | 41 | Home for Sick Children... ... | 204 | 1,562 | 1,868 | 1,047 | 237 | 283 | 1,567 | 1,253 |
| 24 | 20 | General Lying-in... ... ... | 500 | 1,787 | 3,423 | 686 | 2,624 | ... | 3,310 | ... |
| 18 | 15 | Clapham Maternity & Dispensary | 421 | 5,163 | 2,411 | 317 | 1,012 | 818 | 2,147 | ... |
| 54 | 50 | Royal, for Children and Women . | 583 | 8,379 | 4,354 | 2,254 | 964 | 257 | 3,475 | 520 |
| 42 | 33 | Royal Eye ... ... ... ... ... | 632 | 17,529 | 3,341 | 2,317 | 176 | 532 | 3,025 | 295 |
| 11 | 10 | Eltham Cottage ... ... ... | 170 | ... | 725 | 610 | 19 | 165 | 794 | ... |
| 30 | 11 | Beckenham Cottage ... ... | 210 | ... | 912 | 599 | 35 | 133 | 767 | 125 |
| 18 | 12 | Blackheath Cottage ... ... | 156 | 992 | 907 | 763 | 35 | 113 | 911 | ... |
| 32 | 21 | Bromley Cottage ... ... ... | 340 | ... | 1,484 | 933 | 240 | 157 | 1,330 | 540 |
| 14 | 11 | Chislehurst, &c., Cottage ... | 169 | ... | 696 | 489 | 31 | 144 | 664 | ... |
| 12 | 6 | Sidcup Cottage ... ... ... | 137 | 101 | 493 | 385 | 27 | 113 | 525 | ... |
| 7 | 6 | Shortlands Convalescent... ... | 103 | ... | 183 | 180 | 1 | 4 | 185 | ... |
| 16 | 12 | Livingstone Cottage ... ... | 132 | ... | 1,030 | 884 | 9 | 55 | 948 | ... |
| 10 | 8 | Woolwich and Plumstead Cottage | 85 | 15 | 544 | 351 | 24 | 105 | 480 | ... |
| 1,935 | 1,453 | | 20,412 | 251,757 | 217,409 | 46,083 | 93,770 | 8,148 | 148,001 | 46,923 |
| | | DISPENSARIES. | | | | | | | | |
| ... | ... | Battersea Provident ... ... | ... | 19,828 | 3,372 | 101 | 61 | 3,260 | 3,422 | ... |
| ... | ... | Brixton, etc. ... ... ... | ... | 3,570 | 624 | 561 | 34 | 63 | 658 | ... |
| ... | ... | Camberwell Provident ... ... | ... | 8,783 | 1,845 | 335 | 192 | 1,426 | 1,953 | 200 |
| ... | ... | Clapham ... ... ... ... | ... | 1,391 | 333 | 227 | 21 | 113 | 361 | 450 |
| ... | ... | Deptford Medical Mission ... | ... | 2,267 | 345 | 280 | 31 | 62 | 373 | ... |
| ... | ... | East Dulwich Provident ... | ... | 4,616 | 840 | 106 | 2 | 759 | 867 | ... |
| ... | ... | Forest Hill ... ... ... ... | ... | 2,743 | 677 | 228 | ... | 471 | 699 | ... |
| ... | ... | Greenwich Provident ... ... | ... | 2,889 | 626 | 76 | 10 | 561 | 647 | ... |
| ... | ... | Royal South London ... ... | ... | 4,332 | 649 | 508 | 113 | ... | 621 | ... |
| ... | ... | South Lambeth, etc. ... ... | ... | 1,953 | 509 | 337 | 11 | 158 | 506 | 150 |
| ... | ... | Walworth Provident ... ... | ... | 767 | 282 | 40 | 32 | 156 | 228 | ... |
| ... | ... | Wandsworth Common ... ... | ... | 995 | 211 | 45 | ... | 166 | 211 | ... |
| ... | ... | Woolwich, &c., Provident ... | ... | 1,734 | 388 | 44 | ... | 279 | 323 | ... |
| 1,935 | 1,453 | | 20,412 | 307,625 | 228,110 | 48,971 | 94,277 | 15,622 | 158,870 | 47,723 |

### WESTMINSTER DISTRICT.—Comprising Westminster City and Liberties.

| No. of Beds. | No. of Beds Daily Occupied. | HOSPITALS. | In-patients. | Out-patients. | Total Expenditure. | Charitable. | Proprietary. | Patients' Payments. | Total Income. | Legacies not included in previous column. |
|---|---|---|---|---|---|---|---|---|---|---|
| | | | | | £ | £ | £ | £ | £ | £ |
| 175 | 147 | Charing Cross ... ... ... | 2,248 | 19,168 | 18,699 | 9,919 | 1,586 | 186 | 11,691 | 5,954 |
| 223 | 184 | King's College ... ... ... | 2,551 | 19,193 | 18,934 | 9,964 | 2,279 | 227 | 12,470 | 4,936 |
| 202 | 162 | Westminster ... ... ... ... | 2,363 | 22,254 | 24,192 | 6,149 | 2,888 | ... | 9,037 | 5,636 |
| 152 | 139 | Ventnor, for Consumption ... | 755 | ... | 15,539 | 6,669 | 2,108 | 3,992 | 12,769 | 478 |
| 22 | 17 | Grosvenor, for Women & Children | 188 | 3,434 | 2,167 | 1,323 | 13 | 542 | 1,878 | 300 |
| 64 | 49 | Hospital for Women ... ... | 738 | 4,490 | 6,226 | 3,165 | 347 | 484 | 3,996 | 640 |
| 25 | 8 | National, for Diseases of Heart... | 65 | 2,040 | 2,151 | 1,202 | 82 | 501 | 1,785 | ... |
| 36 | 26 | Royal Westminster Ophthalmic... | 676 | 10,215 | 2,381 | 2,021 | 677 | 56 | 2,754 | 327 |
| 50 | 46 | Royal Orthopædic ... ... | 202 | 901 | 2,947 | 1,328 | 448 | 188 | 1,964 | 1,450 |
| 9 | 7 | Royal Ear ... ... ... ... | 240 | 1,989 | 783 | 224 | ... | 548 | 772 | 50 |
| ... | ... | Dental ... ... ... ... ... | ... | 35,687 | 2,029 | 2,661 | 266 | 230 | 3,157 | 60 |
| 27 | 20 | Gordon, for Fistula ... ... | 289 | 918 | 1,809 | 364 | ... | 1,409 | 1,773 | ... |
| 27 | 21 | St. Peter's, for Stone ... ... | 548 | 4,387 | 4,091 | 1,087 | 361 | 2,183 | 3,631 | ... |
| 50 | 37 | St. John's, for Skin ... ... | 242 | 7,539 | 3,910 | 1,300 | ... | 2,309 | 3,609 | 320 |
| 1,062 | 863 | | 11,105 | 132,215 | 105,858 | 47,376 | 11,055 | 12,855 | 71,286 | 20,151 |
| | | DISPENSARIES. | | | | | | | | |
| ... | ... | Public ... ... ... ... ... | ... | 2,741 | 740 | 365 | 191 | ... | 556 | 1,178 |
| ... | ... | St. George and St. James ... | ... | 3,467 | 526 | 431 | 8 | 30 | 469 | ... |
| ... | ... | St. George's, Hanover Square ... | ... | 1,926 | 490 | 387 | ... | 131 | 518 | ... |
| ... | ... | Western ... ... ... ... ... | ... | 11,154 | 1,530 | 395 | 428 | 715 | 1,538 | 100 |
| ... | ... | Westminster General ... ... | ... | 6,091 | 623 | 326 | 166 | 122 | 614 | ... |
| 1,062 | 863 | | 11,105 | 157,594 | 109,767 | 49,280 | 11,848 | 13,853 | 74,981 | 21,429 |

### ST. MARYLEBONE AND WEST CENTRAL DISTRICT.

Comprising St. Marylebone, St. John's Wood, Bloomsbury, Holborn, etc.

| No. of Beds. | No. of Beds Daily Occupied. | HOSPITALS. | In-patients. | Out-patients. | Total Expenditure. | Income. | | | Total Income. | Legacies not included in previous column |
|---|---|---|---|---|---|---|---|---|---|---|
| | | | | | | Charitable. | Proprietary. | Patients' Payments. | | |
| | | | | | £ | £ | £ | £ | £ | £ |
| 70 | 44 | French ... ... ... ... | 875 | 3,611 | 3,600 | 4,000 | 109 | ... | 4,109 | 1,398 |
| 50 | 16 | Italian ... ... ... ... | 215 | 4,732 | 2,207 | 1,611 | 218 | ... | 1,829 | 100 |
| 103 | 73 | London Homœopathic ... ... | 1,128 | 21,014 | 10,132 | 4,515 | 3,620 | 906 | 9,041 | 853 |
| 51 | 26 | SS. John and Elizabeth ... ... | 57 | ... | 2,148 | 955 | 825 | ... | 1,780 | 90 |
| 381 | 334 | The Middlesex ... ... ... | 3,758 | 48,911 | 40,075 | 12,413 | 7,855 | ... | 20,268 | 15,216 |
| 68 | 44 | Alexandra for Children ... ... | 101 | 380 | 3,417 | 3,463 | 132 | 276 | 3,871 | ... |
| 30 | 28 | Hospital for Incurable Children | 34 | ... | 1,304 | 423 | 97 | 454 | 974 | 270 |
| 238 | 143 | Hospital for Sick Children ... | 1,844 | 19,837 | 19,141 | 19,500 | 4,263 | ... | 23,763 | 2,481 |
| 20 | 14 | British Lying-in ... ... ... | 354 | 529 | 2,438 | 640 | 1,971 | 34 | 2,645 | ... |
| 74 | 55 | Queen Charlotte's Lying-In ... | 1,321 | 971 | 4,928 | 3,451 | 317 | 106 | 3,874 | 3,693 |
| 50 | 43 | New Hospital for Women ... | 604 | 14,296 | 5,408 | 3,262 | 585 | 1,622 | 5,469 | 240 |
| 49 | 41 | Samaritan Free ... ... ... | 471 | 7,824 | 5,827 | 6,217 | 220 | ... | 6,437 | 1,377 |
| 200 | 183 | National for the Paralysed, &c.... | 895 | 5,959 | 16,631 | 3,147 | 1,883 | 2,839 | 7,869 | 4,519 |
| 25 | 9 | Hospital for Epilepsy, &c. ... | 46 | 748 | 1,488 | 699 | 46 | 346 | 1,091 | 5,367 |
| 50 | 36 | West End, for Epilepsy, &c. ... | 316 | 3,378 | 3,443 | 2,660 | 77 | 522 | 3,259 | 430 |
| 28 | 16 | Central London Ophthalmic ... | 338 | 12,538 | 1,786 | 1,673 | 26 | ... | 1,699 | ... |
| 13 | 6 | Western Ophthalmic ... ... | 156 | 10,154 | 966 | 497 | 144 | ... | 641 | ... |
| 60 | 51 | National Orthopædic ... ... | 211 | 721 | 2,503 | 1,337 | 22 | 878 | 2,237 | ... |
| 20 | 13 | Establishment for Gentlewomen. | 164 | ... | 2,424 | 711 | 173 | 937 | 1,821 | 500 |
| ... | ... | National Dental ... ... ... | ... | 23,835 | 1,325 | 383 | ... | 935 | 1,318 | ... |
| 16 | 11 | London Throat ... ... ... | 665 | 5,140 | 1,410 | 387 | ... | 1,077 | 1,464 | ... |
| 8 | 2 | Metropolitan Ear, Nose & Throat | 125 | 3,379 | 445 | 152 | ... | 210 | 362 | ... |
| 1,604 | 1,188 | | 13,678 | 187,957 | 133,056 | 72,096 | 22,583 | 11,142 | 105,821 | 36,534 |
| | | DISPENSARIES. | | | | | | | | |
| ... | ... | Bloomsbury Provident ... ... | ... | 807 | 282 | 40 | ... | 221 | 261 | ... |
| ... | ... | London Medical Mission ... | ... | 7,062 | 1,348 | 896 | 58 | 263 | 1,217 | ... |
| ... | ... | Infirmary for Consumption ... | ... | 372 | 502 | 165 | 186 | ... | 351 | 50 |
| ... | ... | Portland Town ... ... ... | ... | 1,348 | 170 | 189 | 4 | 13 | 206 | ... |
| ... | ... | St. John's Wood Provident ... | ... | 5,660 | 779 | 282 | 2 | 425 | 709 | ... |
| ... | ... | St. Marylebone General... ... | ... | 3,537 | 775 | 410 | 163 | 199 | 772 | ... |
| ... | ... | Western General... ... ... | ... | 16,072 | 1,347 | 1,146 | 23 | 59 | 1,228 | 200 |
| 1,604 | 1,188 | | 13,687 | 222,815 | 138,259 | 75,224 | 23,019 | 12,322 | 110,565 | 36,784 |

### STRATFORD AND EAST-END DISTRICT.

Comprising Bethnal Green, Tower Hamlets, West Ham, Whitechapel, Hackney, Stepney, Limehouse, Poplar, and the East.

| No. of Beds. | No. of Beds Daily Occupied. | HOSPITALS. | In-patients. | Out-patients. | Total Expenditure. | Income. | | | Total Income. | Legacies not included in previous column. |
|---|---|---|---|---|---|---|---|---|---|---|
| | | | | | | Charitable. | Proprietary. | Patients' Payments. | | |
| | | | | | £ | £ | £ | £ | £ | £ |
| 130 | 109 | German ... ... ... ... | 1,559 | 22,802 | 9,944 | 5,845 | 2,381 | 456 | 8,682 | 424 |
| 767 | 657 | London ... ... ... ... | 12,154 | 161,762 | 102,551 | 48,488 | 26,475 | 1,375 | 76,338 | 15,335 |
| 50 | 32 | Mildmay Mission Hospital ... | 468 | 8,030 | 3,412 | 2,602 | 587 | 45 | 3,234 | ... |
| 71 | 50 | Poplar ... ... ... ... | 954 | 23,742 | 7,619 | 7,782 | 897 | 131 | 8,810 | 625 |
| 60 | 42 | West Ham, &c. ... ... ... | 684 | 22,836 | 6,320 | 4,675 | 200 | ... | 4,875 | 1,062 |
| 34 | 24 | Walthamstow, &c. ... ... | 364 | 1,061 | 1,582 | 1,259 | 453 | ... | 1,712 | 136 |
| 164 | 116 | City of London for Dis. of the Chest | 832 | 9,281 | 11,428 | 8,550 | 294 | ... | 8,844 | 6,380 |
| 135 | 108 | East London for Children ... | 1,857 | 36,324 | 10,089 | 9,585 | 1,087 | ... | 10,672 | 630 |
| 38 | 32 | St. Mary's, Plaistow ... ... | 700 | 16,754 | 3,299 | 3,016 | 166 | 127 | 3,309 | 100 |
| 18 | 10 | East End Mothers' Home ... | 248 | 286 | 1,658 | 1,020 | 504 | 49 | 1,573 | 770 |
| 8 | 5 | Passmore Edwards Cottage, T'lb'ry | 74 | 516 | 598 | 659 | 23 | ... | 682 | ... |
| 13 | 8 | Canning Town Cottage ... ... | 140 | 1,162 | 936 | 627 | 288 | 162 | 1,077 | ... |
| 1,488 | 1,193 | | 20,034 | 304,556 | 159,436 | 94,108 | 33,355 | 2,345 | 129,808 | 25,462 |
| | | DISPENSARIES. | | | | | | | | |
| ... | ... | Eastern ... ... ... ... ... | ... | 5,697 | 935 | 545 | 278 | 186 | 1,009 | ... |
| ... | ... | Hackney Provident ... ... | ... | 927 | 258 | 65 | ... | 192 | 257 | ... |
| ... | ... | London ... ... ... ... ... | ... | 2,308 | 517 | 142 | 279 | ... | 421 | ... |
| ... | ... | Queen Adelaide's... ... ... | ... | 5,943 | 493 | 333 | 219 | ... | 552 | ... |
| ... | ... | Tower Hamlets ... ... ... | ... | 4,244 | 783 | 808 | 22 | 170 | 1,000 | ... |
| ... | ... | Whitechapel Provident ... ... | ... | 4,826 | 862 | 75 | ... | 770 | 845 | ... |
| ... | ... | Leman Street, Provident ... | ... | 1,311 | 633 | 204 | ... | 325 | 529 | ... |
| 1,488 | 1,193 | | 20,034 | 329,812 | 163,917 | 96,280 | 34,153 | 3,988 | 134,421 | 25,462 |

### ISLINGTON AND NORTH-WEST DISTRICT.

Comprising Islington, Holloway, Highbury, Hampstead, Highgate, St. Pancras, Stoke Newington, Tottenham, &c.

| No. of Beds. | No. of Beds Daily Occupied. | HOSPITALS. | In-patients. | Out-patients. | Total Expenditure. | Income. Charitable. | Income. Proprietary. | Income. Patients' Payments. | Total Income. | Legacies not included in previous column. |
|---|---|---|---|---|---|---|---|---|---|---|
| | | | | | £ | £ | £ | £ | £ | £ |
| 159 | 143 | Great Northern Central ... ... | 2,034 | 31,361 | 13,048 | 7,013 | 1,155 | 610 | 8,778 | 2,210 |
| 32 | 20 | Hampstead Hospitul ... ... | 312 | 830 | 2,959 | 3,436 | 13 | 234 | 3,683 | 500 |
| 120 | 81 | London Temperance ... ... | 1,282 | 22,339 | 10,934 | 5,956 | 1,242 | 298 | 7,496 | 7,122 |
| 53 | 33 | North-West London ... ... | 494 | 15,019 | 5,135 | 2,430 | 377 | 25 | 2,832 | 1,133 |
| 73 | 39 | Tottenham Training ... ... | 628 | 11,634 | 4,894 | 3,597 | 19 | 67 | 3,683 | 127 |
| 185 | 162 | University College ... ... | 2,696 | 37,453 | 20,371 | 8,912 | 2,953 | 21 | 11,886 | 7,570 |
| 80 | 77 | North London Consumption ... | 493 | 3,839 | 7,768 | 6,124 | 141 | 10 | 6,275 | 1,079 |
| 200 | 67 | London Fever ... ... ... | 643 | ... | 13,925 | 7,786 | 1,768 | 1,618 | 11,172 | 1,478 |
| 28 | 15 | Invalid Asylum ... ... ... | 212 | ... | 809 | 436 | 135 | 155 | 726 | ... |
| 14 | 12 | Children's Home Hospital, Barnet | 51 | ... | 569 | 438 | 11 | 64 | 513 | ... |
| 14 | 12 | Enfield Cottage ... ... ... | 158 | ... | 655 | 441 | 39 | 183 | 663 | ... |
| 30 | 21 | Memorial Cottage, Mildmay ... | 184 | ... | 1,522 | 517 | 1,087 | 129 | 1,733 | ... |
| 27 | 14 | St. Saviour's Home ... ... | 111 | ... | 2,159 | 987 | 17 | 640 | 1,644 | 500 |
| 42 | 32 | Friedenheim Home ... ... | 139 | ... | 3,419 | 2,569 | 125 | 186 | 2,880 | 180 |
| 30 | 23 | St. Monica's, Brondesbury ... | 44 | ... | 1,524 | 811 | 205 | 326 | 1,342 | ... |
| 25 | 16 | Willesden Cottage ... ... | 211 | ... | 1,113 | 1,179 | 60 | 82 | 1,321 | ... |
| 20 | 7 | Bushey Heath Cottage ... ... | 88 | 27 | 968 | 690 | 61 | 122 | 873 | ... |
| 1,132 | 774 | | 9,780 | 122,502 | 91,772 | 53,322 | 9,408 | 4,770 | 67,500 | 21,899 |
| | | DISPENSARIES. | | | | | | | | |
| ... | ... | Camden Provident ... ... | ... | 1,078 | 282 | 19 | ... | 250 | 269 | ... |
| ... | ... | Hampstead Provident ... ... | ... | 11,423 | 990 | 214 | 27 | 760 | 1,001 | ... |
| ... | ... | Holloway and North Islington ... | ... | 3,503 | 826 | 288 | 42 | 320 | 650 | ... |
| ... | ... | Islington ... ... ... ... | ... | 11,937 | 963 | 282 | 19 | 560 | 861 | ... |
| ... | ... | St. Pancras and Northern ... | ... | 1,429 | 519 | 229 | 130 | 93 | 452 | ... |
| ... | ... | Stamford Hill, &c. ... ... | ... | 6,066 | 748 | 604 | 162 | ... | 766 | ... |
| 1,132 | 774 | | 9,780 | 157,938 | 96,100 | 54,958 | 9,788 | 6,753 | 71,499 | 21,899 |

### KENSINGTON AND WEST DISTRICT.

Comprising Kensington, Paddington, Bayswater, Kilburn, Chelsea, Brompton, Fulham, Hammersmith, Chiswick, Brentford, Acton, Ealing, etc.

| No. of Beds. | No. of Beds Daily Occupied. | HOSPITALS. | In-patients. | Out-patients. | Total Expenditure. | Income. Charitable. | Income. Proprietary. | Income. Patients' Payments. | Total Income. | Legacies not included in previous column. |
|---|---|---|---|---|---|---|---|---|---|---|
| | | | | | £ | £ | £ | £ | £ | £ |
| 351 | 297 | St. George's ... ... ... | 3,975 | 25,643 | 47,432 | 12,192 | 15,301 | ... | 27,493 | 11,974 |
| 281 | 253 | St. Mary's ... ... ... ... | 3,826 | 40,291 | 30,460 | 11,607 | 2,886 | ... | 14,493 | 7,850 |
| 133 | 124 | West London ... ... ... | 1,846 | 28,708 | 8,961 | 6,541 | 303 | ... | 6,844 | 2,962 |
| 321 | 272 | Hospital for Consumption ... | 1,425 | 7,415 | 36,223 | 14,500 | 8,018 | ... | 22,518 | 14,100 |
| 16 | 14 | Belgrave, for Children ... ... | 250 | 5,085 | 1,231 | 761 | 161 | ... | 922 | 200 |
| 50 | 49 | Cheyne, for Sick & Incurable Chldn | 20 | ... | 2,540 | 1,913 | 465 | 357 | 2,735 | 2,150 |
| 46 | 36 | Paddington Green, for Children | 574 | 14,322 | 3,821 | 2,934 | 194 | 307 | 3,435 | 794 |
| 92 | 78 | Victoria, for Children ... ... | 953 | 16,967 | 6,745 | 5,132 | 480 | 365 | 5,977 | 30 |
| 52 | 43 | Chelsea, for Women ... ... | 673 | 2,636 | 5,704 | 3,976 | 180 | 801 | 4,957 | 709 |
| 105 | 88 | Cancer ... ... ... ... | 725 | 1,640 | 13,711 | 5,666 | 3,345 | ... | 9,011 | 7,485 |
| 135 | 83 | Female Lock ... ... ... | 627 | ... | 4,962 | 2,564 | 28 | 1,784 | 4,376 | 100 |
| 16 | 8 | Epsom and Ewell Cottage ... | 98 | ... | 1,010 | 668 | 12 | 207 | 887 | ... |
| 30 | 13 | Reigate and Redhill Cottage ... | 196 | ... | 1,027 | 815 | 31 | 108 | 954 | ... |
| 16 | 9 | Wimbledon Cottage ... ... | 136 | ... | 673 | 648 | 11 | 111 | 770 | ... |
| 15 | 8 | Hounslow Cottage ... ... | 312 | 478 | 540 | 447 | 204 | 47 | 698 | 32 |
| 1,659 | 1,375 | | 15,636 | 143,185 | 165,040 | 70,364 | 31,619 | 4,087 | 106,070 | 48,386 |
| | | DISPENSARIES. | | | | | | | | |
| ... | ... | Brompton Provident ... ... | ... | 794 | 377 | 112 | 81 | 190 | 383 | ... |
| ... | ... | Chelsea, &c. ... ... ... | ... | 3,894 | 621 | 394 | 174 | ... | 568 | ... |
| ... | ... | Chelsea Provident ... ... | ... | 559 | 247 | 29 | 17 | 157 | 203 | ... |
| ... | ... | Kensal Town Provident ... ... | ... | 713 | 314 | 35 | ... | 261 | 296 | ... |
| ... | ... | Kensington ... ... ... | ... | 3,212 | 767 | 796 | 65 | ... | 861 | 500 |
| ... | ... | Kilburn, Maida Vale ... ... | ... | 2,303 | 472 | 368 | 43 | ... | 411 | 50 |
| ... | ... | Kilburn Provident ... ... | ... | 4,442 | 1,169 | 65 | 13 | 1,119 | 1,197 | ... |
| ... | ... | Notting Hill Provident ... ... | ... | 472 | 218 | 54 | 7 | 105 | 166 | ... |
| ... | ... | Paddington Provident ... ... | ... | 3,111 | 553 | 152 | 26 | 353 | 531 | ... |
| ... | ... | Royal Pimlico Provident... ... | ... | 1,219 | 937 | 360 | 14 | 573 | 947 | ... |
| ... | ... | Westbourne Provident ... ... | ... | 1,523 | 427 | 49 | 38 | 352 | 439 | ... |
| 1,659 | 1,375 | | 15,636 | 165,427 | 171,142 | 72,778 | 32,097 | 7,197 | 112,072 | 48,936 |

### CITY AND EAST CENTRAL DISTRICT.

Comprising the City, St. Luke's, Shoreditch, Finsbury, and Clerkenwell.

| No of Beds. | No. of Beds Daily Occupied. | HOSPITALS. | In-patients. | Out-patients. | Total Expenditure. | Income. | | | Total Income. | Legacies not included in previous column. |
|---|---|---|---|---|---|---|---|---|---|---|
| | | | | | | Charitable. | Proprietary. | Patients' Payments. | | |
| | | | | | £ | £ | £ | £ | £ | £ |
| 112 | 79 | Metropolitan ... ... ... | 964 | 33,708 | 11,216 | 9,151 | 435 | 1,441 | 11,027 | 1,405 |
| 170 | 120 | Royal Free ... ... ... | 1,937 | 32,592 | 11,589 | 5,732 | 1,063 | ... | 6,795 | 3,527 |
| 80 | 61 | Royal, for Diseases of the Chest. | 702 | 6,558 | 7,702 | 6,550 | 147 | ... | 6,697 | 669 |
| 57 | 51 | North-Eastern, for Children ... | 778 | 18,264 | 6,197 | 5,137 | 376 | 711 | 6,224 | 60 |
| 42 | 27 | City of London Lying-In ... | 599 | 1,753 | 4,374 | 950 | 3,752 | 4 | 4,706 | ... |
| 45 | 31 | St. Mark's, for Fistula ... ... | 451 | 1,171 | 4,500 | 1,392 | 731 | ... | 2,123 | 135 |
| 138 | 64 | Royal London Ophthalmic ... | 1,871 | 36,932 | 10,786 | 7,992 | 681 | ... | 8,673 | 1,097 |
| 45 | 45 | City Orthopædic ... ... ... | 290 | 3,358 | 3,039 | 1,730 | 39 | ... | 1,769 | 100 |
| 17 | 9 | Central London Throat and Ear . | 240 | 8,428 | 2,239 | 1,620 | 77 | 1,521 | 3,218 | ... |
| 706 | 487 | | 7,832 | 142,764 | 61,642 | 40,254 | 7,301 | 3,677 | 51,232 | 6,993 |
| | | DISPENSARIES. | | | | | | | | |
| ... | ... | City ... ... ... ... ... | ... | 5,570 | 1,755 | 1,189 | 147 | ... | 1,336 | ... |
| ... | ... | City of London and East London | ... | 24,480 | 1,666 | 95 | 84 | 2,034 | 2,213 | ... |
| ... | ... | Farringdon General ... ... | ... | 2,746 | 632 | 338 | ... | 209 | 547 | ... |
| ... | ... | Finsbury ... ... ... ... | ... | 13,045 | 899 | 630 | 168 | 262 | 1,060 | ... |
| ... | ... | Metropolitan ... ... ... | ... | 5,451 | 843 | 405 | 122 | 265 | 792 | ... |
| ... | ... | Royal Genera ... ... ... | ... | 2,971 | 764 | 314 | 294 | 79 | 687 | ... |
| 706 | 487 | | 7,832 | 197,027 | 68,201 | 43,225 | 8,116 | 6,526 | 57,867 | 6,993 |

### THE METROPOLITAN HOSPITALS.—A SUMMARY OF THE WORK DONE IN 1900.

It will be seen from the following summary that the Voluntary Hospitals and medical Charities of London, during the twelve months ending December 31st, 1900, relieved over One million six hundred thousand patients at a cost of £975,496.

| No. of Beds. | No. of Beds Daily Occupied. | HOSPITALS AND DISPENSARIES. | In-patients. | Out-patients. | Total Expenditure. | Income. | | | Total Income. | Legacies not included in previous column. |
|---|---|---|---|---|---|---|---|---|---|---|
| | | | | | | Charitable. | Proprietary. | Patients' Payments. | | |
| | | | | | £ | £ | £ | £ | £ | £ |
| 1,935 | 1,453 | Newington and South District ... | 20,412 | 307,625 | 228,110 | 48,971 | 94,277 | 15,622 | 158,870 | 47,723 |
| 706 | 487 | City and East Central District... | 7,832 | 197,027 | 68,201 | 43,225 | 8,116 | 6,526 | 57,867 | 6,993 |
| 1,062 | 863 | Westminster District ... ... | 11,105 | 157,594 | 109,767 | 49,280 | 11,848 | 13,853 | 74,981 | 21,429 |
| 1,604 | 1,188 | St. Marylebone and West Central District ... ... ... | 13,678 | 222,815 | 138,259 | 75,224 | 23,019 | 12,322 | 110,565 | 36,784 |
| 1,659 | 1,375 | Kensington and West District ... | 15,636 | 165,427 | 171,142 | 72,778 | 32,097 | 7,197 | 112,072 | 48,936 |
| 1,132 | 774 | Islington & North-West District | 9,780 | 157,938 | 96,100 | 54,958 | 9,788 | 6,753 | 71,499 | 21,899 |
| 1,488 | 1,193 | Stratford and East-End District | 20,034 | 329,812 | 163,917 | 96,280 | 34,153 | 3,988 | 134,421 | 25,462 |
| 9,586 | 7,333 | | 98,477 | 1,538,238 | 975,496 | 440,716 | 213,298 | 66,261 | 720,275 | 209,226 |

The Ordinary Income only amounted to £720,275, leaving a deficiency of £255,221 on the year's work. The legacies received in 1900 amounted to £209,226, being £69,950 less than the amount received in 1899. **It therefore appears that the actual nett deficiency for the year 1900, when the whole of the legacies received have been credited to income, amounts to £45,995.**

### HEALING THE SICK.

In this great City of London the work of healing the sick is always going on, and to the army of those who give of their substance to aid the hospitals fresh recruits are constantly being added. So great is the sympathy with illness and suffering that even a larger number of helpers would be forthcoming if it were possible to direct the attention more easily to the work going on daily in some large hospital near at hand. But for the majority of people, and especially those who live in the wealthier and suburban quarters of London, the object lesson of a hospital is too far away to excite individual interest. The public may want to give, but amongst so many hospitals it is difficult to make a selection. These institutions do not minister to one particular locality, as do the hospitals in the Provinces; they all for the most part receive inmates from every quarter of the Metropolis, from the suburbs and from the country beyond, and from all these parts all these hospitals ought to receive support.

It is, however, by no means easy for those who are willing to support the hospitals to make up their mind to which hospital they should give, and it is here that the Hospital Sunday Fund becomes so useful. Not only is it a means through which all may safely dispense their charity, but it is especially a means through which those who have neither time or opportunity for making up their minds among the many hospitals around them may make their contributions with full confidence that they will be carefully distributed among thoroughly deserving charities.

## Figures and Tables

**Figure f1:**
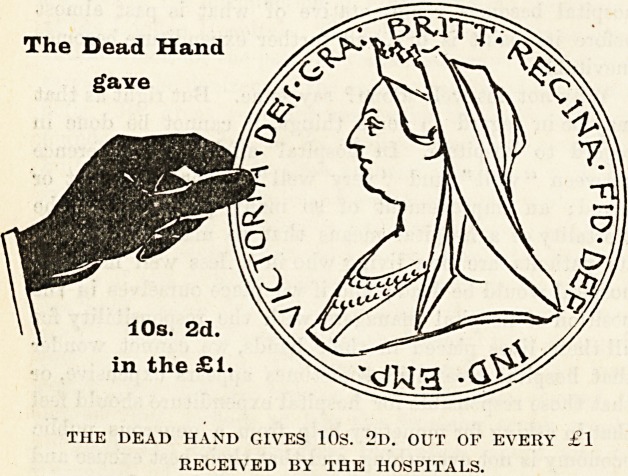


**Figure f2:**
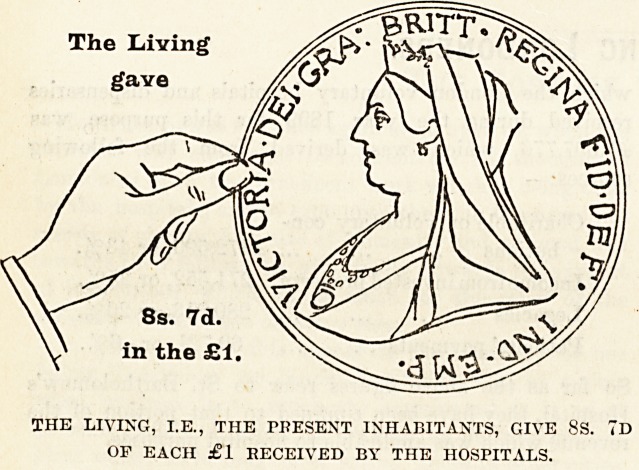


**Figure f3:**